# Supplementary material for: Environmental factors and occurrence of horseshoe crabs in the northcentral Gulf of Mexico
Source: PLoS One. 2021 Jan 4;16(1):e0243478. doi: 10.1371/journal.pone.0243478 (PMC7781375; doi:10.1371/journal.pone.0243478)
Supplement: S1 Table — (DOCX) [file pone.0243478.s001.docx]

Horseshoe crab field surveys and associated environmental data for 2012 and 2013 in the north central Gulf of Mexico are available from the Dauphin Island Sea Laboratory Data Management Center, <https://www.disl.org/research/data-management-center>

S1 Table. Kappa statistic (Kappa = observed agreement - expected agreement / 1 – expected agreement) for live horseshoe crabs and LCLU agreement.

|  | HSC | LCLU | Totals | % |
| --- | --- | --- | --- | --- |
| Suitable | 49 | 2 | 51 | 87 |
| Not Suitable | 3 | 5 | 8 | 13 |
| Totals | 52 | 7 | 59 |  |
| % | 88 | 12 |  |  |

Using the data in Table S1, the first term needed in the Kappa formula is “observed agreement”. Observed agreement is the addition of the top left and lower right cells in the table (49 + 5), which represents horseshoe crabs found in suitable and unsuitable habitats as predicted.

Observed agreement = 54/59 = 0.92

To find “expected or chance agreement”, multiply the percentages of column 1 by row 1 and column 2 by row 2.

Expected agreement = (0.88 * 0.87) + (0.12 * 0.13) = 0.79

Kappa Formula: (0.92 – 0.79) / (1 – 0.79) = 0.62
