# Supplementary figures and images for: Environmental factors and occurrence of horseshoe crabs in the northcentral Gulf of Mexico
Source: PLoS One. 2021 Jan 4;16(1):e0243478. doi: 10.1371/journal.pone.0243478 (PMC7781375; doi:10.1371/journal.pone.0243478)

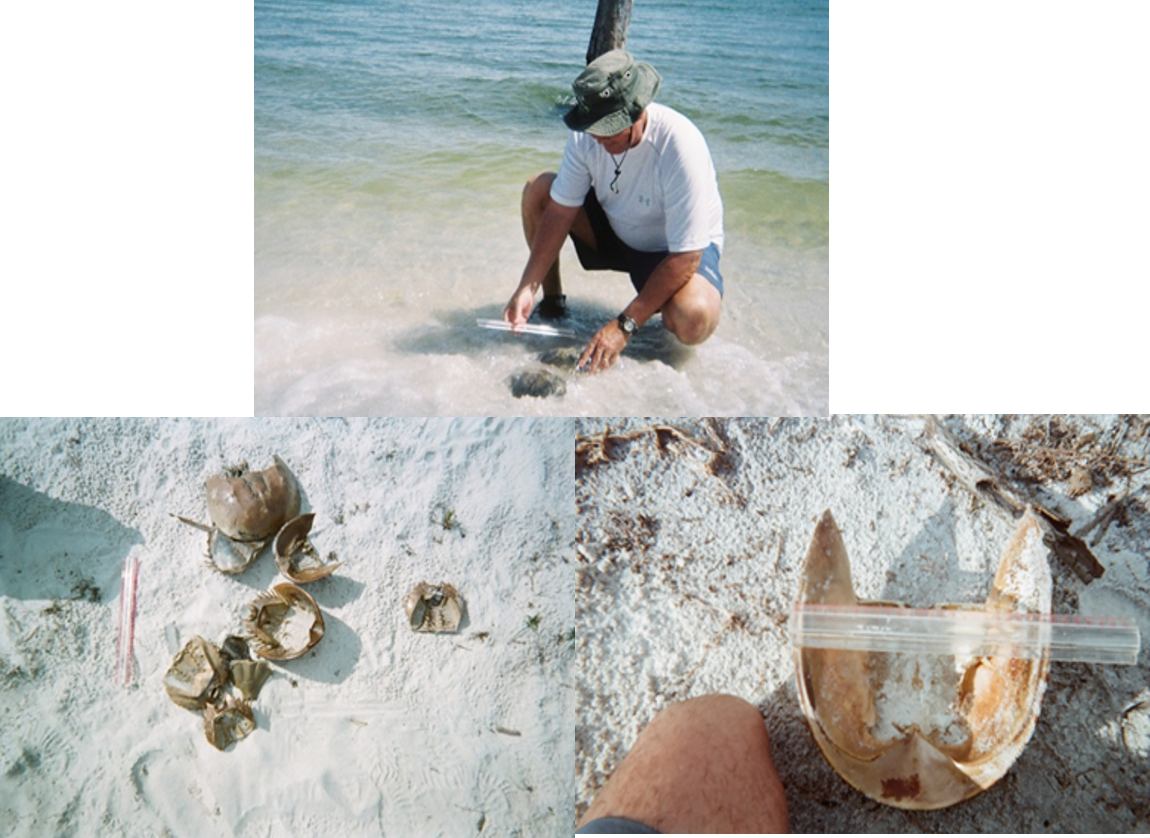

Supplement: S1 Fig — Measuring carcasses, molts (top) and live horseshoe crabs (bottom; amplexed pair) to determine prosomal width. (TIF) [file pone.0243478.s002.tif]
