# Supplementary material for: Environmental factors and occurrence of horseshoe crabs in the northcentral Gulf of Mexico
Source: PLoS One. 2021 Jan 4;16(1):e0243478. doi: 10.1371/journal.pone.0243478 (PMC7781375; doi:10.1371/journal.pone.0243478)
Supplement: Attachment — Submitted filename: Rebuttal_letter-submit.docx [file pone.0243478.s003.docx]

João Miguel Dias, Ph.D.
Academic Editor
PLOS ONE

22 October 2020

Dear Dias,

Thank you for the opportunity to submit a revised version of our manuscript, *Environmental factors and occurrence of horseshoe crabs in the northcentral Gulf of Mexico*. We are pleased with the positive nature of the comments and think that the reviewer’s suggestions have improved the manuscript and increased its value. We also thank you for the allowance of an extension to complete these revisions during our unusually active hurricane season.

Below, we respond point-by-point, to the comments of the reviewers. We hope that you and the reviewers find the revised manuscript suitable for publication in *PLOS ONE*.

None of this material has been published or is under consideration elsewhere. Thank you for your time and consideration and please do not hesitate to contact us if you have any questions.

Sincerely,

Maury Estes

Maurice G. Estes, Jr., Ph.D.

Associate Program Manager for NASA Ecological Forecasting Program

Research Scientist VI

University of Alabama in Huntsville

Maury.estes@nsstc.uah.edu

256-468-1154 cell

**Response to editorial and reviewers’ comments to the Author**

Reviewer #1

1. What is the evidence that these animals are rare?

**We do not claim horseshoe crabs are rare. The abundance of horseshoe crabs in the northern Gulf of Mexico is unknown. We do know, based on our own observations and the existing literature, that populations are less dense (sparse) compared to populations in the eastern Gulf of Mexico and on the U.S. Atlantic coast, as discussed in the introduction. We took care to use the term ‘sparse’, rather than ‘rare’ throughout the text for this reason. In this revised version, we additionally replaced the term ‘sparse’ with ‘less densely populated’ in some parts of the text for clarity and we confirmed that the term ‘rare’ is not in the text.**

2. Surveys conducted May – Sep, do not include the previously estimated period of peak of spawning during late April and May. Hence, populations may not be sparse.

**While the reviewer is correct that our surveys did not cover a portion of the spawning period that may include a second (or in this case, a first peak), it is important to note that this study is not a spawning survey and not intended to focus primarily on spawning habitat (many of this reviewers comments seem to derive from a spawning-focused lens). The difference in horseshoe crab density at the northcentral Gulf of Mexico sites we studied (including the data from Fulford and Haehn noted by the reviewer) indicate the density of horseshoe crabs is so much lower at these sites than at sites in peninsular Florida and the mid-Atlantic, that inclusion of these time periods could not possibly change the description of these populations from ‘sparse’ in comparison. The Rudloe data cited by the reviewer make this same point, and these data were collected from peninsular Florida, which has a higher density of crabs.**

3. Data on size is confounded by not separating males and females for live animals and carcasses.

**This is a good point, and we have included these values in the text to show the data separated by sex. Thank you for this suggestion.**

4. Rudloe 1980 found that water inundation (tide + wind surge) was the most important influence on horseshoe crab numbers in the northeastern Gulf, but the present study does not collect data on actual water levels.

**Rudloe 1980 is one of the foundational spawning population studies in peninsular Florida. That work was done to define conditions of spawning. Since that time the relationship between numbers of spawning crabs and water height has been very well documented, and sampling protocols have been developed to normalize for this variable. Although we were not targeting spawning animals, to maximize likelihood of finding animals at survey sites, we used these established protocols (sampling within 2 hours of high tide) to normalize for water height. For this reason, we could focus on other factors that varied among sites as is now common practice.**

5. Averaging by month or year does not consider zeros in the data; the data on numbers of individuals almost certainly included many zeros …, which means that a different kind of analysis is needed for data of this type.

**The reviewer is correct that there are zeros in the dataset as can be seen in the table and figures, but we made certain that all statistical tests we applied were suitable to our dataset prior to analyses, and we did not violate any assumptions of those tests.**

6. The molt transport analysis may have incorrectly calculated the % recovery.

**The % recovery is accurately reported as 2.5%. Five molts were recovered out of 195 marked and placed in the field (not 130 as assumed by the reviewer); molts were placed 3 times at n = 65 each time. Thank you for checking on this calculation.**

7. The LCLU is the strongest part of the paper and should be emphasized.

**We thank the reviewer for the kind words and agree that this portion of the study is very important. The preceding components in the manuscript are needed to provide context for these analyses. To highlight the importance of this work, however, we gave this aspect of the study a priority position in the discussion.**

8. Additional comments
a. Line 32. The abstract begins with a bit of hyperbole: “This study provides the first regional-scale data…” It is not the first because Fulford and Haehn have data for this region.

**Fulford and Haehn data cover only spawning animals on two islands in Mississippi. This study spans a much larger spatial area, roughly 55 km along MS and AL coasts of the northcentral Gulf of Mexico. The Fulford and Haehn study also did not collect comprehensive environmental data nor make comparisons to the timing and location of the animals, limiting the application of those data to understand habitat suitability without collection and analyses of additional data. Hence, this study is the first to make this effort. We do not, however, think this is a major point and have removed ‘first’ from the abstract.**

b. Line 62. “Numbers of horseshoe crabs are reportedly higher to the east along the Florida panhandle…” this would be a good point to reference Rudloe 1980.

**Reference added**

c. Line 77. “… in the northwestern Gulf of Mexico”, except that as you know they are found in microtidal environments.

**While horseshoe crabs are found in microtidal environments within range of apex populations, their numbers decline moving into these habitats. For this reason and other evidence associated with evolutionary-scale changes in geomorphology, the lack of tidal magnitude is the prevailing theory for modern horseshoe crabs not being found in the northwestern Gulf of Mexico (refer to citations in the text and several easily discoverable publications by the late Dr. Carl Shuster and/or Dr. Robert Loveland). Note, we also included the statement that this idea “may explain” the distribution because this idea is a theory.**

d. Line 89. “…where horseshoe crab populations are already sparse and…” the population density in this area is not actually known

**See comments above under #1 and #2.**

e. Line 105 -114. This paragraph is all about remote sensing. Is this a study using remote sensing?

**Yes, the study uses remotely sensed data for the land cover land use analysis (C-CAP Landsat derived) and NLDAS data for precipitation, wind speed and wind direction as noted in the methods. This point is an important component of the manuscript and is also addressed in the discussion because of the potential benefit these data provide to supplement and enhance *in situ* data collection.**

f. Line 119 sentence ending, “and most adults thought to migrate from shelf or offshore waters seasonally….” needs a citation.

**Reference added: Shuster and Botton (1985), A contribution to the population biology of horseshoe crabs Limulus polyphemus in Delaware Bay. *Estuaries* 8:363–372**

g. Line 172. Should be “… discharge in winter and spring than in summer and fall…”

**Thank you for catching this type-error; “is” replaced with “in”.**

h. Line 186. Should be “…Surveys were conducted from May to August at biweekly and…”

**“typically” deleted.**

i. Lines 189-192. When exactly did the surveys take place at each site? Fig. 5 presents the same data just for live animals, but does not separate the sites; Is effort taken into account?

**This figure has been edited to clarify numbers at each site, including sites not surveyed for a given date (now indicated by an X in new figure 4). On some dates surveys were missed due to weather conditions, especially late in the summer of 2012 when one hurricane and numerous thunderstorms made small boat travel unsafe. The pattern of occurrence (shown in Fig. 3) did not change when normalized to sampling effort among sites. Hence, level of effort is not the driver of the observed data differences. To clarify this point, the level of effort (occurrence per day) was added to Fig. 3.**

j. Lines 192-194. …there are 13 different dates or data points to compare with the environmental data. Is this a sufficient sample size to come to conclusions such as horseshoe crab “…presence…[is] mediated by a combination of distance from areas of high freshwater discharge…”

**There were a sufficient number of samples to detect patterns in the data and perform statistical analyses that yielded valid outcomes.** **While a larger sample size is always desirable, we had sufficient sampling during the varying weather conditions at the 4 sites to allow comparisons. Given the variation in environmental conditions and among sites, including variation in discharge, our reported findings on horseshoe crab occurrence and relationships to environmental attributes are reasonably established. As noted by the reviewer, these data are hard won and will always have some limitations. The text has been edited throughout out to clarify that our results are based on the period of study and with an eye to clarifying the potential limitations of the interpretation.**

k. Lines 80-90. Of course a paper can’t cite all relevant publications, but this discussion of the factors influencing horseshoe crab distributions does not reference a number of highly relevant and directly related studies (e.g. Vasquez et al., Cheng et al.)

**The suggested references have been added.**

l. Line 207. Should be “…Calipers were used for smaller juvenile molts to determine prosomal width…”

**“molts” added to statement as noted.**

m. Line 247. These are climatic means for Dauphin Island.

**The reviewer is correct, these values are climatic means for Dauphin Island, which are provided as a proxy for climate conditions in the study area. Comparable climate data were not available for the other 3 sites surveyed. For clarity, this table was moved to the results section and the comparison was integrated into the results section.**

n. Line 252-267. How does this give the height of the ocean floor, relative to what?

**A DEM is a 3D representation of land or subsurface elevation values to represent terrain or bathymetric with the dataset referenced. Acoustic techniques such as echo sounders are often used to obtain points or values in marine regions. The Vdatum bathymetry values can to be related to various tidal datums. The bathymetry values used in this study are related to mean high water.**

o. Line 289-290. “…to constitute an area large enough that correlations with numbers of live horseshoe crabs, molts or carcasses would not be random.” What is that area? A reference is needed here.

**The C-CAP land cover land use data (LCLU) is at 30 m native resolution (30 m x 30 m grid), which was only a 30 m shoreline length and very fragmented with other lclu classes. These data were resampled to coarser resolutions of 100 m and 300 m to identify clusters of LCLU classes that could reasonable represent major shoreline habitat characteristics. 300 m was selected as noted in the manuscript (300 m x 300 m grid), which represents a shoreline length of about 300 m per LCLU class. Reference added for the C-CAP data.**

p. Line 292. Fig. 2. The LCLU classes are a valuable part of this study... Do all the classes occur at the four sites? The figure legend is not clear.

**The figure was edited to make the shorelines more visible and clarify the caption relative to horseshoe crab habitat. Not all of the classes occur on the four sites. Given the narrow width of some of the sites (Dauphin and Petit Bois Islands) the same pixel and class may be in one grid cell that covers both north and south shorelines.**

q. Line 359. Should be “…Islands, and during 2013 99% of all juvenile molts were found on Petit Bois Island…”

**We reworded the sentence to maintain the comma after 2013, which is grammatically necessary.**

r. Line 362. Table 2. It is not clear what the wind direction and wind speed numbers refer to. … taking means of directions is problematic since 359 is right next to 01. Circular distributions require different stats (not arithmetic mean).

**We thank the reviewer for pointing out the need to clarify the methods used to calculate wind direction. The methods have been revised in the text. Wind speed was considered as the force of the wind during the time surveys were conducted for that site. Wind direction is the direction (0-360 degrees) from which the wind is blowing (e.g. 180° would represent winds traveling from the south to the north). The reviewer is correct that we needed to (and did) calculate the mean of circular quantities. Briefly, the means of the cosines and sines of each angle (wind direction during a survey) were computed. The sum of the cosine and sines was divided by the number of surveys (n). The ratios of the sum of the sines to sum of the cosines was computed and the arctangent of this value was considered the mean angle of the wind direction in degrees.**

s. Line 396, 402-404. “… placement, and as of 2019 no additional tagged molts were recovered (Fig 6).” This point is not illustrated with Fig. 6. Maybe Fig. 8?

**Our apologies. In the previous version of the text some figures were mis-labeled. We thank the reviewer for pointing out this error, which has been fixed in the revised text.**

t. Line 404. Should be “… counted on beach surveys were correctly associated with the site where they were found.”

**Statement edited to add “where they were” as noted.**

u. Line 407; line 415. It would be much easier to compare these data with other populations if they were separated into males and females.

**See response to #3 above.**

v. Line 416. Not sure how these peaks are determined in Fig. 6. Should these be presented as frequencies instead of raw numbers – the legend says frequency but the axis says number?

**The figure is a histogram showing the frequency distribution of horseshoe crabs at sizes depicted in the bins on the x-axis. Mixed distribution analyses were previously performed on these size classes as described in Estes et al. (2015) and cited in the text; this study compares newly collected data to those established size distributions.**

w. Line 420. Size data are in Fig. 6 not 7. The adult size data need to be separated by sex.

**See comments to #3 and #8s above.**

x. Line 449; Fig. 9., Since daily discharge numbers are not related to daily or weekly horseshoe crab numbers, I don’t think this figure is particularly useful. The central point is that the two years differ, which is given in the text with numbers.

**Additional comparisons on horseshoe crab occurrence, size and sex ratios are in the revised text. The discharge data are directly related to the years and time periods that were surveyed. Without the figure, the pattern of discharge fluctuations when surveys were conducted could not be visualized. Also, the figure highlights (along with the text numbers) the magnitude and temporal fluctuations of discharge across the two years of study.**

y. Line 464. Should be “The number of live horseshoe crabs was significantly related…”

**“were” changed to “was”**

z. Line 503. “Accordingly, Petit Bois Island, which is more remote from Mobile Bay…” might be better to say, “Consistent with this view”.

**Text was changed as suggested.**

aa. Line 509. “toward the limit of their distribution in Mississippi.” Should be Louisiana since they are found in Chandeleur Islands.

**“Mississippi” changed to “Louisiana”**

bb. Line 553. “… has limited reproductive value [3].” Might be better to say limited value in population recruitment.

**“reproductive value” deleted and “population recruitment” added.**

cc. Line 557. … adult sizes are consistent with some regions, but how do they compare with areas elsewhere in the Gulf of Mexico or elsewhere in their distribution?

**The sizes are comparable with other horseshoe crabs found in MS and LA barrier islands (Fulford and Haehn) as well as the northern and western Gulf coast of Florida. Text was added to make these demographic comparisons. It is noted, however, that Fulford and Haehn and Rudloe targeted spawning animals (we did not) and in some cases reported data in ways that make direct comparison difficult.**

dd. Line 583. Should be “In this case, just two years of data increased our knowledge of horseshoe crab habitat use…” since Fulford & Haehn was a very fine study.

**Text was edited to delete “where limited or no prior studies had been conducted”. While the Fulford and Haehn study is complementary to this work, it is a very different study in scope; limited in spatial scale and to spawning animals only, without regard to comprehensive environmental conditions.**

ee. Line 622. …this study contributes to a growing body of evidence that conservation efforts for horseshoe crabs will benefit from considering local habitat attributes …” citations to a few of these other studies are needed.

**This sentence is the concluding sentence to an entire paragraph dedicated to this point; note all of the citations throughout the paragraph. In addition, we added Botton et al. (2018), which also specifies geomorphology on a local scale is detectable and can mediate horseshoe crab occurrence.**

9. Problems with references (incorrect citations)
   **Citations corrected**
10. Missing references: Rudloe, A. 1980.; Cheng et al. 2016; Vasquez et al. 2017

**References added**

Reviewer #2

This is a well conducted study...and a well written manuscript. Horseshoe crabs are of such great interest to those who study the ecology of northern Gulf barrier island shorelines. This manuscript provides valuable information which expands current knowledge of barrier island ecology in the northcentral Gulf of Mexico. The research plan was well designed. The field work and data analyses were well executed. This is an interesting study worthy of publication.

**We thank this reviewer for the kind words and recognition of the value of the dataset.**

**Additional authors’ revisions:** In addition to the suggested revisions by the reviewers, we copyedited the manuscript to correct some minor type errors (spelling, extra spaces, etc.), consistently referred to the region as northcentral GOM (rather than nGOM) for clarity, and made some modifications to abbreviations and rounding in the Tables for consistency and clarity. We also updated the figure numbers and in-text references to the figures and tables. These minor changes are not tracked in the text to avoid confusion with reviewer suggestions.
